# Quality of Life and Stress Levels in Patients under Home Mechanical Ventilation: What Can We Do to Improve Functioning Patients at Home? A Survey Study

**DOI:** 10.3390/ijerph20010874

**Published:** 2023-01-03

**Authors:** Magdalena Kwiatosz-Muc, Bożena Kopacz, Anna Fijałkowska-Nestorowicz

**Affiliations:** 1 1st Clinic of Anaesthesiology and Intensive Therapy, Medical University of Lublin, Jaczewskiego Street 8, 20-093 Lublin, Poland; 2 Department of Anaesthesiological and Intensive Care Nursing, Medical University of Lublin, Chodźki Street 7, 20-093 Lublin, Poland

**Keywords:** home mechanical ventilation, quality of life

## Abstract

Background: Home mechanical ventilation (HMV) is becoming more widely available in many countries. Objectives: The aim of this study was to measure the health-related quality of life and stress levels of patients ventilated mechanically at home. The relation between quality of life and stress levels was investigated including multiple regression analysis. Methods: 100 patients treated with HMV in Poland were surveyed with the WHOQOL-BREF questionnaire and Perceived Stress Scale (PSS-10). Results: 26% of patients assessed their quality of life as bad or very bad and 34% as good or very good. Stress levels measured with PSS-10 Scale were high level. For the group of patients with neurological disorders, stress levels were significantly higher than for the group of patients with pulmonological disorders. Conclusions: The higher the stress levels of patients, the lower the quality of life in particular domains. Improving the living conditions of HMV patients can influence improving their quality of life.

## 1. Background

Home mechanical ventilation (HMV) is currently becoming more widely available in many countries [1]. It is a complex and multidisciplinary treatment for patients with symptoms of chronic respiratory insufficiency due to pulmonary disorders, scoliosis or neuromuscular disorders. Treating these patients with home mechanical ventilation programs decreases the frequency of visits in emergency departments and hospitalizations in general [2].

An increasing number of patients included in HMV care have been under observation for many years [3,4,5,6]. Several studies have shown increased survival among patients undergoing this treatment [7]. Recent guidelines of a number of scientific associations recommend the use of home mechanical ventilation for patients with symptoms of chronic respiratory insufficiency [8,9,10,11,12]. HMV seems to improve health-related quality of life [13,14].

Patients ventilated mechanically at home are dependent on the correct functioning of medical equipment. Despite the fact that patients stay in their own environment, among relatives, they could suffer from a number of psychological phenomena such as anxiety, feelings of dependence, limitations in communication and stress [15]. The experiences of caregivers of patients undergoing HMV have been described in the literature [16,17] more widely than the experiences of the patients themselves [18,19,20] also in regard to health-related quality of life [21,22,23]. Health-related quality of life (HRQOL) is a particularly important outcome of treating patients suffering from chronic diseases.

The aim of this study was to measure the health-related quality of life and stress levels of patients ventilated mechanically at home. The relation between quality of life and stress levels was also investigated as well as the regression model explaining quality of life.

## 2. Study and Methods

### 2.1. Sample Selection

The study was designed as unicentric questionnaire survey. Patients treated with HMV in the southeast of Poland, were invited to participate in the study in a non-random, consecutive and voluntary way. Only adult patients were invited to participate in the study with the length of HMV treatment between 6 months and 10 years. The Ethics Committee of the Medical University of Lublin approved the study. All participants gave their consent. A total of 125 surveys were distributed by the members of the research team to the caregivers of the patients in the period of 1 July 2017 to 28 February 2018. Surveys were left and completed without the presence of the member of therapeutic team. The survey in the envelope was taken at the next visit. Finally, the envelope with the survey was passed to the research team. The traditional form of a paper and pencil survey was used. Participants had no time limitation to fill out the surveys.

### 2.2. Methods

The World Health Organisation Quality of Life-BREF Assessment Instrument (WHOQOL-BREF) questionnaire was employed to measure health related quality of life and Perceived Stress Scale (PSS-10) was used to assess stress levels. The relevant sociological and demographic data as well as medical history were surveyed by the authors.

The WHOQOL-BREF questionnaire is a universal research tool to investigate health-related quality of life [24]. It was adapted to Polish conditions by Wołowicka and Jaracz [25] and is recognized as a reliable tool to assess the quality of life of adult people [24,26,27,28]. The original questionnaire is available at the WHO website [29]. It is composed of 26 questions concerning four domains of life: somatic, psychological, social and environmental. In the somatic domain (DOM 1), everyday activities, dependency on treatment, energy, feeling of being tired, mobility, feeling discomfort, quality of rest and sleeping and the ability to work are assessed. In the psychological domain (DOM 2), one’s own reflections concerning their physical appearance, the presence and intensification of positive and negative feelings, self-assessment, spiritualism, religiosity, personal faith, thinking, learning and concentration are assessed. In the social domain (DOM 3), personal relations, the support of relatives and sexual activity are assessed. The environmental domain (DOM 4) assesses living and financial conditions, the availability and quality of medical services, the possibility of recreation, transport and one’s feeling of safety. There are two questions that are analysed separately: Question 1 concerns the general perception of quality of life (“How would you rate your quality of life?”) and question 2 concerns an individual’s perception of their own health (“How satisfied are you with your health?”). Answers for the questions are constructed on a 5-point Likert scale. Basic values in particular domains are transferred to a 0–100 scale following WHO guidelines [30].

PSS-10 is a simple research tool used to assess stress levels over the previous month [31,32]. It consists of 10 statements which refer to personal impressions of feeling troubled and ways of behaving in a difficult situation. The person examined agrees or disagrees with the statements on a 5-point scale. The final result of the scale is the sum of all points (up to 40). This sum is then converted into a 10-point standard sten score. Results from 1 to 3 sten scores are related to low levels of stress. Results between 7 and 10 sten scores are related to high levels of stress. Cronbach’s alpha for the Polish version of the PSS-10 Scale has a range from 0.72 to 0.92 [33].

### 2.3. Statistical Methods

The data were analysed statistically using the STATISTICA 13.3 program (Tibco Software Inc., Palo Alto, CA, USA). Qualitative variables were characterized by multiplicity and percentage, while quantitative variables were characterized by basic classical statistical measures: mean and standard deviation. In the case of WHOQOL-BREF, as the manual suggests, there are minimum and maximum measures as well. The scope of minimal and maximal were given. The Shapiro–Wilk test was used to examine the compliance of the distribution of features with the normal distribution. In order to examine the significance of the differences between the median values, parametric tests were used. The T-Student test was used to compare two autonomous trials, and mono-factor ANOVA variance analysis was used when comparing a larger number of means. The F test was used to verify the homogeneity of variances for two groups, and Levene’s test was preferred for more groups. The Rank U Mann–Whitney test was used to examine the significance of differences between two autonomous variables, and the Wilcoxon test for two related samples and the ANOVA Kruskal–Wallis test for a larger number of independent samples were used. Dependence analysis between two qualitative variables was based on Spearman’s coefficient (R) and the t test was used for the significance of this coefficient in the population. Spearman’s correlation coefficient was used for the correlation between stress levels and quality of life measured with the WHOQOL-BREF questionnaire. The test of different significance levels between two structure indicators was used to compare differences between structure indicators (percent). Simple and multiple regression models in the group N explaining quality of life were developed. A *p*-value < 0.05 was considered as statistically significant.

## 3. Results

The 125 surveys were distributed among HMV patients, and 100 correctly completed surveys were analysed (filled out in accordance with the instructions and containing no missing items). The mean age in the examined population was 66 (Q1–Q3; 57–72). The examined population included 71 (71%) patients with pulmonary diseases. Among these patients, 65 were diagnosed with chronic obstructive pulmonary disease (COPD), 2 with bronchial asthma, 2 with apnea syndrome, 1 person with amyloidosis and 1 with pulmonary emphysema. Twenty-nine (29%) from the entire population examined had respiratory insufficiency due to neurological disorders such as amyotrophic lateral sclerosis (ALS) (13 people), Duchenne’s dystrophy (DMD) (6 people), myasthenia gravis (4 people), sclerosis multiplex (SM) (2 people), cerebral palsy (2 people), central core disease (1 person) and Arnold–Chiarie Syndrome (1 person). The examined population was divided into two groups: group P comprised 71 patients with pulmonary diseases, and group N comprised of 29 patients with neuromuscular disorders. Twenty-nine patients from the examined population were treated with invasive ventilation (IV). Patients in the N group were significantly younger than patients in the P group (*p* < 0.001). The characteristics of the examined population are shown in detail in Table 1. Mean time of duration of HMV was 3.1 years in group N and 2.0 years in group P. There was no statistically significant difference in duration of ventilation between these groups (Table 2).

### 3.1. Quality of Life Assessed with WHOQOL-BREF

Twenty-six percent of patients in the entire population assessed their quality of life as bad or very bad. Thirty-four percent of patients in the entire population assessed their quality of life as good or very good, and forty percent of respondents assessed their general quality of life as ordinary. Fifty-eight percent of all respondents were not satisfied with their health status, seventeen percent were satisfied, and twenty-five percent were neither satisfied nor dissatisfied with their health status. Answers for question 1 of WHOQOL-BREF (concerning general perception of quality of life) and question 2 (individual’s perception of one’s own health) are detailed in Table 3. We can see that patients with neurological disorders assessed their quality of life as bad or very bad (*p* = 0.030) significantly more often than patients with pulmonological disorders. They were also significantly more often dissatisfied with their quality of life than patients with pulmonological disorders (*p* = 0.012). Patients with pulmonological disorders assessed their quality of life as ordinary (*p* = 0.0125) more often than patients with neurological disorders.

The results for the examined population in regard to the WHOQOL-BREF domains can be found in Table 4. The highest result was in the environmental domain, while the lowest was in the somatic domain. There are no statistically significant differences among domains between groups N and P. There is, however, a tendency for patients with pulmonological disorders (*p* = 0.090 < 0.1) to assess social relations (social domain) more positively than patients with neurological disorders (Table 4).

General quality of life (answers for question 1) and general health self-assessment (answers for question 2) in the examined population (n = 100) and their relation with chosen demographic features were examined. For age, sex, marital status, level of education and place of residence, no statistically significant relation was found. A statistically significant difference in assessing general quality of life (answers for question 1) between patients in different living conditions was found. The rank comparison multiple test for pairs of preferable groups shows only a tendency (*p* = 0.055) towards significant difference in satisfaction with health status (answers for question 2) between groups of patients living in very good conditions and those living in bad and very bad conditions.

Quality of life in the four domains in the examined population (n = 100) in relation to chosen demographic features was assessed. Men assessed their quality of life in the somatic domain more positively than women (*p* = 0.037). Married people assessed their quality of life in terms of social relations more positively than unmarried people (social domain, *p* = 0.035). Respondents in very good living conditions assessed their quality of life in the psychological dimension as better than respondents in bad and very bad living conditions. Respondents in very good living conditions assessed their quality of life in social terms better than respondents in worse (bad and very bad) living conditions. Concerning the environmental domain of the quality of life, statistically significant differences among groups of patients with different living conditions were discovered. Quality of life in particular domains in the examined population, depending on chosen demographic features, is shown in Table 5.

Chronbach’s coefficient for WHOQOL-BREF in our study was in total α = 0.918 (DOM 1 α = 0.740, DOM 2 α = 0.868, DOM 3 α = 0.601, DOM 4 α = 0.746).

### 3.2. Stress Level Assessment with PSS-10 Scale

Stress levels measured with the PSS-10 Scale were related to seven sten scores, which indicates high levels of stress. Chronbach’s coefficient for PSS-10 in our study was α = 0.86. For the group of patients with neurological disorders, stress levels measured with PSS-10 were significantly higher than for the group of patients with pulmonological disorders (Mann–Whitney U test, Z = 2.499, *p* = 0.012). However, the patients in the N group were significantly younger than patients in the P group (*p* < 0.001), no significant correlation between PSS-10 SUM and age was found.

The relation between perceived stress levels measured with the PSS-10 Scale and different demographical features was investigated. No statistically significant differences in stress levels in demographical feature groups were found (Table 6).

**Figure 1:**
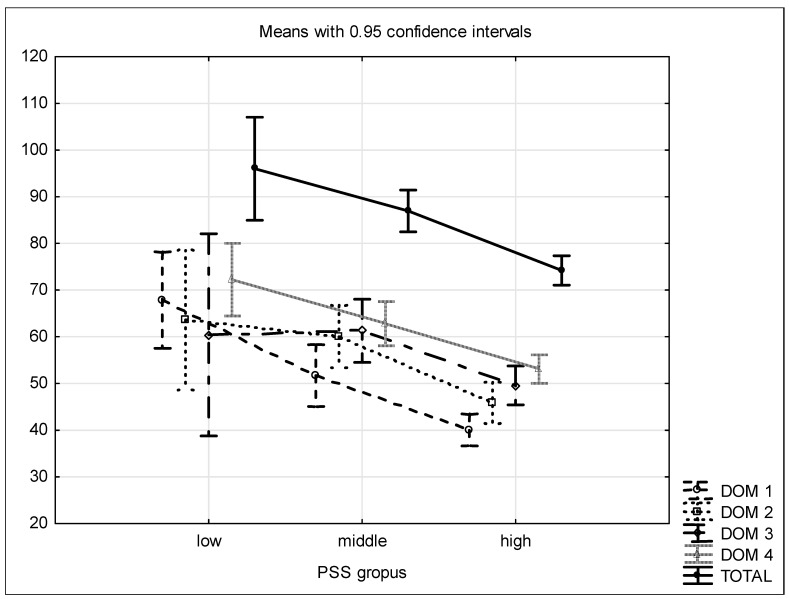
Mean WHOQOL BREF results with 95% of confidence interval in stress level groups.

The relation between perceived stress levels, measured with the PSS-10 Scale, and quality of life, measured with WHOQOL-BREF, was analysed (Figure 1). Significantly strong negative correlations between PSS-10 and quality of life were observed in particular domains (Table 7).

Variance analysis to compare quality of life depending on stress levels was performed. It showed that stress levels influence the quality of life assessment in all domains. Patients with high stress levels (PSS-10 STEN ≥ 7) have significantly lower results in the quality of life assessment in the somatic, psychological and environmental domains than patients with lower stress levels. Concerning the social domain, patients with high stress levels tended to assess quality of life as lower than patients with average stress levels (Scheffe test, *p* = 0.059). Concerning the somatic domain, patients with average stress levels assessed their quality of life as significantly better than patients with low stress levels (Table 8).

### 3.3. Regression Analysis

The simple regression model was developed in the group of patients with neurological diseases. The model explains quality of life assessment in somatic domain (DOM 1) depending on perceived stress level in the PSS-10 Scale:

DOM 1 = −1.909 × PSS_SUM_ + 85.771

The regression model explains 53% (R^2^ = 53%) of changes in quality of life assessment in the somatic domain in this group of patients. Relative estimation error in the somatic domain is 35.6%, which is at an acceptable level. The coefficient of −1.909 for variable PSS_SUM_ indicates that the higher the perceived stress level, the lower the quality of life in the somatic domain in this group. If the PSS_SUM_ goes higher by one unit, quality of life the in somatic domain goes down by 1.909 (Table 9).

In group N, the multiple regression model explaining quality of life assessment in the social domain (DOM 3) depending on perceived stress level in the PSS-10 Scale and living conditions of the patients may be developed:

DOM 3 = −1.321 × PSS_SUM_ + 11.975 × LC − 1136.965

where LC means living conditions.

This regression model explains 73% (R^2^ = 73%) of changes in quality of life assessment in the social domain in group N. The relative estimation error in the social domain in this group based on stress level and living conditions is 14.7%, which shows the high precision of this estimation. When the perceived stress level is higher by one unit level for quality of life, then in the social domain the value is lower in this group by 1.321. Moreover, when the living conditions in the patient’s opinion are evaluated higher by one unit, then the quality of life in the social domain goes higher by 11.975 (Table 10). Perceived stress level has more influence than living conditions on the quality of life in the social domain in group N.

In group N simple regression model explaining quality of life (WHOQOL BREF SUM) depending on perceived stress level (PSS_SUM_):

WHOQOL BREF SUM = −2.034 × PSS_SUM_ + 123.591

This regression model explains 59% (R^2^ = 59%) of changes of quality of life in this group. The relative estimation error for quality of life in group N based on perceived stress level is 14.7%, which shows the high precision of this estimation. When the perceived stress level is higher by one unit, then the quality of life assessment is lower by 2.034 (Table 11).

In group N, no sufficient regression model explaining assessment of quality of life in DOM 2 and DOM 4 was developed. In group P, a regression model sufficiently explaining quality of life in any of the domains was not possible to develop either.

## 4. Discussion

Patients ventilated mechanically in intensive care units (ICU) experience a number of negative psychological phenomena: vulnerability, anxiety, fear and loneliness [34]. Other patients describe suffering from emotional responses such a hopelessness, high level of frustration and stress [35]. Being dependent on health professionals without being able to communicate could be the cause of this phenomena [34]. A Swedish study based on interviews with mechanically ventilated patients in ICU shows that being dependent on other people and technical medical equipment for survival creates a sense of being delivered to the hands of personnel [36]. Having lines and tubes in one’s body was described as stressful [36]. Users of home mechanical ventilation encounter similar psychological challenges. HMV permits increased wellbeing of patients thanks to a home-based lifestyle compared with institutional-based treatment [37]. Nevertheless, HMV is related to a difficult life situation full of psychological, social and existential challenges [38]. HMV users are dependent on care givers and equipment. Some studies report that HMV users can find their health good, whereas healthcare professionals find the opposite [20,39]. Lindahl et al. suggest that HMV results in an increase in the user’s quality of life and normalization of their everyday life. Quality of life is improved in the physical and psychological dimensions [39]. On the other hand, HMV patients have the feeling of being trapped and describe constant feelings of fearfulness and worry. They find their homes have become institutionalized [39].

The quality of life of patients treated with HMV is rarely investigated and has not been examined in Poland. In this study, 40% of respondents assessed their quality of life as average. Thirty-four percent of interviewees assessed their quality of life as good or very good. Compared with a healthy population living in an industrial area in Poland, examined in a similar period of time with the same instrument (WHOQOL-BREF) by Szemik et al. [40], the quality of life of respondents in our study was much lower. The mean results in particular domains in the Szemik et al. study ranged from 62 to 75, depending on the domain. Healthy people had the highest results in social domains, while our respondents had the highest results in the environmental domain.

Kaub-Wittemer et al. [41] found that quality of life among SLA patients undergoing HMV was average. The Munich Quality of Life Dimensions List (MQLDL), used in the German study, investigates quality of life in four dimensions (psychological, social, physical and daily). In this study, patients had better quality of life in the social dimension than the patients in our study. Respondents in our study had higher quality of life in the environmental domain (which is equivalent to the daily dimension in MQLDL).

The present study showed that patients with neurological disorders assessed their quality of life in the social domain more positively than patients with pulmonological disorders (Table 4). This is different from other results, specifically Huttman et al. [22], who measured quality of life of HMV patients with the SRI Questionnaire (Severe Respiratory Insufficiency Questionnaire). This instrument measures the quality of life of patients in seven dimensions. Social functioning is equivalent to the social domain in WHOQOOL-BREF. In Huttman et al.’s study, the group of patients with neurological disorders showed lower quality of life in the social functioning dimension than patients with pulmonological disorders [22].

The seldom described experiences of patients undergoing HMV shows that this treatment causes distress and anxiety in patients’ lives [42] as they have a constant feeling of dependence. In Schaepe and Ewers’ study in Germany [43], one of the respondents described the total necessity of trusting one’s nurse, which reflects this high feeling of dependence. Similar feelings of patients concerning their caregivers were described in a Scottish study [44]. Difficulties in communication and the frustration with the loss of the possibility of speech is described as a relevant negative impression of HMV users [45]. In the present study, we focused on one aspect of the psychological functioning of the examined patients—stress levels. Data concerning stress levels in patients undergoing HMV are insufficient. High stress levels among patients in this study are, to some extent, coherent with the results of other studies, which describe feelings of uncertainty, distress and dependence on others.

Psychological stressors perceived by mechanically ventilated patients in the ICU are described as intensive care unit environmental factors such as dyspnea, fear, anxiety and pain. Patient education and sharing information may be helpful in reducing stress [46]. Some studies showed that listening to music may have beneficial effects on the anxiety of mechanically ventilated patients [47]. Music therapy can lead to relaxation and anxiety reduction in mechanically ventilated patients [48,49]. We did not investigate this aspect in the present study. Stress coping styles among HMV patients could be an area of interest in our future research. Investigating the resistance and resilience strategies, patient education and music therapy could be used to improve patients’ quality of life. It would be useful to further investigate the differences in possible different strategies for improving the quality of life of patients ventilated at home taking into consideration the special needs of both investigated groups in this study. This could be an area of future research as well.

## 5. Study Limitations

The study has several limitations.

Firstly, the number of patients recruited is relatively low. The process of recruiting patients to the study was not randomized and the respondents live in one region of the country. This could be a limitation to the generalization of the results.

Secondly, the group of patients with pulmonological disorders was significantly larger than the group of patients with neurological disorders. It is the result of sample selection. Patients were invited to participate in the study in a non-random, consecutive and voluntary way.

The group of patients with pulmonological disorders was also significantly older than the group of patients with neurological disorders. Stress level, however, is not correlated significantly with age, like quality of life, in particular domains. Similar differences in age between groups of patients divided by type of disorder have been observed by other authors [22].

Additionally: it is worth mentioning that only one method was used to measure quality of life as well as stress levels in the investigated groups. The quality of life and stress levels in HMV patients need further investigation with an alternative method. We have to remember that in every survey study, the results reflect the perceptions of the participants. It should be taken into consideration in formulating any conclusions in survey studies.

## 6. Conclusions

Patients with neurological disorders undergoing HMV assess their quality of life less positively than patients with pulmonological disorders undergoing HMV. Patients undergoing HMV, who declare worse living conditions, showed lower quality of life than patients in better living conditions. Improving the living conditions of these patients can improve their quality of life.

Patients with neurological disorders undergoing HMV have higher stress levels than patients with pulmonological disorders undergoing HMV. The higher the stress levels of patients are, the lower the quality of life in particular domains. The developed multiple regression model for patients with neurological disorders showed that when the perceived stress level is higher by one unit, then the level of quality of life in the social domain is lower in this group. Moreover, when the living conditions in the patient’s opinion are evaluated to be higher by one unit, then the quality of life in the social domain goes higher. However, the perceived stress level has more influence than living conditions on quality of life in the social domain in group N. Implementing psychological help services and stress prophylaxis could be part of improving the quality of life of HMV patients.

## Figures and Tables

**Table 1:** Groups characteristics.

Variables		N-Group (n = 29)		P-Group (n = 71)		Total (n = 100)	
		n	%	n	%	N	%
Type of ventilation	NIV	8	27	63	89	71	71
	IV	21	73	8	11	29	29
Gender	male	20	69	45	63	65	65
	female	9	31	26	37	35	35
Marital status	married	17	59	47	66	64	64
	unmarried	12	41	24	34	36	36
Place of living	urban	19	66	32	45	51	51
	rural	10	34	39	54	49	49
Education	no/elementary	11	38	25	35	36	36
	vocational	6	21	22	31	28	28
	secondary/higher	12	41	24	34	36	36
Living condition	very good	5	17	19	27	24	24
	good	22	76	44	62	66	66
	difficult	2	7	8	11	10	10
Declared quality of life (question 1)	good (4–5)	11	38	23	32	34	34
	moderate (3)	6	21	34	48	40	40
	bad (1–2)	12	41	14	20	26	26
Age		Me	Q_1_–Q_3_	Me	Q_1_–Q_3_	Me	Q_1_–Q_3_
		52	38–59	68	64–74	66	57–72

NIV—not-invasive ventilation, IV—invasive ventilation, N-group—patients with neuromuscular disorders, P-group—patients with pulmonological disorders.

**Table 2:** Duration of HMV by group.

		Duration of HMV (Years)					Test U Mann-Whitney	
		M	SD	Median	Min–Max	Q_1_–Q_3_	Z	*P*
Group	N (n = 29)	3.1	2.9	1.9	0.5–11.0	1.5–3.3	1.428	0.153
	P (n = 71)	2.0	1.5	1.8	0.3–8.0	0.9–2.5		
	Total	2.3	2.1	1.8	0.3–11.0	0.9–2.7		

N-group—patients with neuromuscular disorders, P-group—patients with pulmonological disorders, M—mean, SD—standard deviation, *p*—*p*-value for Mann–Whitney U.

**Table 3:** Percentage of answers for question 1 and question 2 of WHOQOOL-BREF by patient group.

General Perception of Quality of Life (Q1)	Very Bad %	Bad %	Not Good Nor bad %	Good %	Very Good %
N	17	24	24	28	7
P	3	17	48	29	3
Indyvidual’s perception of health (Q2)	Very unsatisfied	Unsatisfied	Not satisfied nor usatisfied	Satisfied	Very satisfied
N	27	38	14	14	7
P	8	46	30	16	0

N—patients with neuromuscular disorders, P—patients with pulmonary diseases, Q1—question 1, Q2—question 2.

**Table 4:** WHOQOL-BREF results in domains by group of patients.

Domains of Quality of Life	Total		N (n = 29)	P (n = 71)			F Test	Test
	M	SD	M	SD	M	SD	*p*	*P*
DOM 1	44.25	16.43	41.13	17.06	45.52	16.12	0.683	0.227 ^(1)^
DOM 2	49.67	19.22	50.29	23.25	49.41	17.50	0.058	0.838 ^(1)^
DOM 3	52.42	18.59	47.41	21.14	54.46	17.18	-	0.090 ^(2)^
DOM 4	56.22	13.75	54.85	12.90	56.78	14.13	0.602	0.527 ^(1)^

N—patients with neuromuscular disorders, P—patients with pulmonary diseases, DOM 1—somatic domain, DOM 2—psychological domain, DOM 3—social domain, DOM 4—environmental domain. ^(1)^ *t*-test, ^(2)^ Mann–Whitney U.

**Table 5:** WHOQOL-BREF results in domains by chosen demographical features (N = 100).

Demographical Variables	n	Domain							
		Somatic		Psychological		Social		Environmental	
		M ± SD	*p*	M ± SD	*p*	M ± SD	*p*	M ± SD	*p*
Gender			0.037 *		0.645		0.631		0.233
Female	35	39.6 ± 15.5		48.5 ± 18.5		51.2 ± 17.3		55.1 ± 12.5	
Male	65	46.8 ± 16.5		50.3 ± 19.7		53.1 ± 19.4		56.8 ± 14.4	
Age			0.895 ^(2)^		0.512 ^(2)^		0.377 ^(2)^		0.680 ^(2)^
<60 years	31	43.8 ± 17.7		52.6 ± 20.8		51.9 ± 20.7		57.0 ± 14.2	
60–69 years	33	43.4 ± 16.0		49.4 ± 15.5		56.3 ± 13.3		54.5 ± 12.8	
≥70 years	35	45.2 ± 16.2		47.0 ± 21.2		50.2 ± 20.1		57.1 ± 14.6	
Merital status			0.966		0.449		0.035 *		0.584
maried	64	44.2 ± 16.4		48.6 ± 20.4		55.3 ± 18.1		56.8 ± 14.3	
unmarried	36	44.3 ± 16.7		51.6 ± 17.1		47.2 ± 18.5		55.2 ± 12.8	
Education			0.627 ^(2)^		0.930 ^(2)^		0.223 ^(2)^		0.306 ^(2)^
no/elementary	36	43.2 ± 15.9		50.6 ± 18.8		51.4 ± 17.3		54.9 ± 12.6	
vocational	28	46.8 ± 15.3		49.6 ± 18.6		57.4 ± 14.4		54.2 ± 14.1	
higher	36	43.4 ± 17.9		48.8 ± 20.6		49.5 ± 22.1		59.0 ± 14.5	
Place of living			0.867		0.227 ^(1)^		0.641 ^(1)^		0.456
urban	51	44.0 ± 15.8		53.2 ± 20.2		53.1 ± 18.7		57.2 ± 13.4	
rural	49	44.5 ± 17.2		46.0 ± 17.6		51.7 ± 18.6		55.2 ± 14.2	
Living conditions			0.106 ^(3)^		0.005^(3)^ **		0.013 ^(2)^*		<0.001 ^(2)^***
very good	24	47.2 ± 15.8		58.3 ± 20.2 ^a^		61.5 ± 17.3 ^a^		66.1 ± 12.8 ^a^	
good	66	44.5 ± 17.3		48.0 ± 18.0 ^ab^		50.4 ± 18.9 ^b^		55.3 ± 11.7 ^b^	
difficult	10	35.7 ± 7.5		39.6 ± 18.8 ^b^		44.2 ± 11.1 ^b^		38.4 ± 7.2 ^c^	

M—mean, SD—standard deviation, *p*—*p*-value for t test/Mann–Whitney U ^(1)^/ANOVA ^(2)^/Kruskal–Wallis test ^(3)^; ^a,b,c^—post hoc test designation (Scheffe for ANOVA, rank for Kruskal–Wallis); groups without common designation differ significantly; * *p* < 0.05, ** *p* < 0.01, *** *p* < 0.001.

**Table 6:** PSS SUM by chosen demographical features (n = 100).

Demographical Variables	PSS SUM		
	n	M ± SD	*p*
Gender			0.131
Female	35	22.4 ± 4.2	
Male	65	20.6 ± 6.1	
Age			0.227 ^(1)^
<60 years	32	22.2 ± 5.0	
60–69 years	33	20.5 ± 5.2	
>=70 years	35	21.4 ± 6.0	
Merital status			0.906
maried	64	21.4 ± 5.7	
Unmarried	36	20.8 ± 5.4	
Education			0.882 ^(1)^
no/elementary	36	21.2 ± 5.6	
Vocational	28	21.3 ± 5.1	
Higher	36	21.2 ± 6.0	
Place of living			0.309
Urban	51	20.7 ± 5.1	
Rural	49	21.8 ± 6.0	
Living conditions			0.881 ^(1)^
very good	24	20.5 ± 4.9	
Good	66	21.3 ± 5.9	
Difficult	10	22.6 ± 4.8	

M—mean, SD—standard deviation, *p*—*p*-value for Mann–Whitney U test/Kruskal–Wallis test ^(1)^.

**Table 7:** Spearman’s rank correlation between perceived stress level measured by PSS-10 and quality of life measured by WHOQOL-BREF.

Couple of Variables	N	R	*p*
DOM 1 & PSS-10	100	–0.484	<0.0001 **
DOM 2 & PSS-10	100	–0.452	<0.0001 **
DOM 3 & PSS-10	100	–0.338	0.0006 *
DOM 4 & PSS-10	100	–0.431	<0.0001 **

DOM 1—somatic domain, DOM 2—psychological domain, DOM 3—social domain, DOM 4—environmental domain, R—Spearman’s coefficient. * *p* < 0.001, ** *p* < 0.0001.

**Table 8:** Quality of life in particular domains of WHOQOL-BREF by groups of patients with different stress levels.

Domain	STRESS LEVEL (PSS-10)						ANOVA *p*
	Low (1–3 sten)		Medium (4–6 sten)		High(7–10 sten)		
	M	SD	M	SD	M	SD	
DOM 1	67.9 ^a^	12.4	51.7 ^b^	12.9	40.0 ^c^	14.8	<0.001 ***
DOM 2	63.5 ^a^	17.9	60.0 ^a^	13.0	45.8 ^b^	19.2	0.002 **
DOM 3	60.4	25.9	61.3	13.2	49.6	18.1	0.027 *
DOM 4	72.3 ^a^	9.4	62.9 ^a^	9.1	53.0 ^b^	13.4	<0.001 ***

DOM 1—somatic domain, DOM 2—psychological domain, DOM 3—social domain, DOM 4—environmental domain, M—mean, SD—standard deviation, *p*—*p*-value for ANOVA; ^a,b,c^—post hoc designation for Scheffe test; groups without common designation differ significantly. * *p* < 0.05, ** *p* < 0.01, *** *p* < 0.001.

**Table 9:** Regression analysis for dependent variable DOM-1 in Group N (n = 29).

Variable	b	SE b	Beta	SE Beta	t(27)	*P*
Constant			85.771	8.333	10.293	0
PSS SUM	−0.730	0.131	−1.909	0.344	−5.555	0

DOM 1—somatic domain, Group N—patients with neurological disorders, b—unstandardized regression weights, beta—standardized regression weights, SE—statistical error, F(1.27) = 30.854.

**Table 10:** Multiple regression analysis for dependent variable DOM-4 in Group N (n = 29).

Variable	b	SE b	Beta	SE Beta	t(26)	*p*
Constant			−1136.965	278.186	−4.07	0.000
PSS SUM	−0.669	0.103	−1.321	0.204	−6.491	0.000
Living conditions	0.454	0.103	11.975	2.718	4.405	0.000

DOM 4—environmental domain, Group N—patients with neurological disorders, b—unstandardized regression weights, beta—standardized regression weights, SE—statistical error, F(2.26) = 34.879.

**Table 11:** Regression analysis for dependent variable WHOQOL BREF SUM in Group N (n = 29).

Variable	b	SE b	Beta	SE Beta	t(27)	*p*
Constant			123.591	7.839	15.766	0.000
PSS SUM	−0.771	0.123	−2.034	0.323	−6.291	0.000

Group N—patients with neurological disorders, b—unstandardized regression weights, beta—standardized regression weights, SE—statistical error, F(1.27) = 39.574.

## Data Availability

The data that support the findings of this study are available from the corresponding author, M.K.M., upon reasonable request.
